# The development of a new accountability measurement framework and tool for global health initiatives

**DOI:** 10.1093/heapol/czz170

**Published:** 2020-06-03

**Authors:** Adriane Martin Hilber, Patricia Doherty, Andrea Nove, Rachel Cullen, Tunde Segun, Sarah Bandali

**Affiliations:** 1 Swiss Tropical and Public Health Institute, Socinstrasse 57, P.O. Box. 4002 Basel, Switzerland; 2 University of Basel, Petersplatz 1, 4001 Basel, Switzerland; 3 Novametrics Ltd, Duffield, Belper, Derbyshire, England DE56 4HQ, UK; 4 Options Consultancy Services Ltd, St Magnus House, 3 Lower Thames Street, London EC3R 6HD, UK; 5 Marie Stopes International, 1 Conway Street, London W1T 6LP, UK

**Keywords:** Accountability, measurement, monitoring, evaluating, global health

## Abstract

The Global Strategy for Women’s Children’s and Adolescents’ Health emphasizes accountability as essential to ensure that decision-makers have the information required to meet the health needs of their populations and stresses the importance of tracking resources, results, and rights to see ‘what works, what needs improvement and what requires increased attention’. However, results from accountability initiatives are mixed and there is a lack of broadly applicable, validated tools for planning, monitoring and evaluating accountability interventions. This article documents an effort to transform accountability markers—including political will, leadership and the monitor–review–act cycle—into a measurement tool that can be used prospectively or retrospectively to plan, monitor and evaluate accountability initiatives. It describes the development process behind the tool including the literature review, framework development and subsequent building of the measurement tool itself. It also examines feedback on the tool from a panel of global experts and the results of a pilot test conducted in Bauchi and Gombe states in Nigeria. The results demonstrate that the tool is an effective aid for accountability initiatives to reflect on their own progress and provides a useful structure for future planning, monitoring and evaluation. The tool can be applied and adapted to other accountability mechanisms working in global health.

**Key Messages**

- Results from accountability initiatives are mixed and often unevaluated, with a lack of broadly applicable tools for planning and evaluating interventions.
- This article introduces a framework and tool for monitoring and evaluating accountability initiatives in global health based on piloting results from Nigeria.

## Introduction

In recent years, accountability has gained prominence as a key strategy to drive progress on the sustainable development goals, through a strong emphasis on tracking progress, reviewing achievements against targets and acting to implement remedial actions. The Global Strategy for Women’s Children’s and Adolescents’ Health emphasizes accountability as essential to ensure that decision-makers have the information required to meet the health needs of their populations and stresses the importance of tracking resources, results and rights to see ‘what works, what needs improvement and what requires increased attention’ (Every Woman Every Child, 2015). The Unified Accountability Framework (UAF), which accompanies the Global Strategy, aims to co-ordinate and support accountability at national level, by bringing together diverse stakeholders and streamlining the monitor, review and act elements of accountability at all levels (EWEC, 2017). A recent commentary in the *Lancet* notes that efforts to advance universal health coverage (UHC) and other global health agendas have recognized the need to have a framework for accountability to advance their agenda (Yamin and Mason, 2019).

Despite this increasing interest, accountability initiatives are often abstract and complex, making them sometimes difficult to comprehend and implement. It is also challenging to measure the extent to which the initiatives actually increase accountability and their contribution towards other observed outcomes and impacts. There is limited understanding of what processes lead to results and under what conditions public officials and those in power will respond to accountability efforts (Nove *et al.*, 2019). To understand how accountability works, there is a need for more monitoring and evaluation around the accountability process itself, and those working in/with accountability to engage in more self-reflection on what results they contribute to, how and why progress is (or is not) made towards increased accountability (Anselmi *et al.*, 2017; de Kok *et al.*, 2017).

Throughout the article, we refer to accountability mechanisms—by which we mean the formalized structures and bodies that operate the monitor, review and act parts of the accountability cycle including the notion of redress or sanction. Accountability mechanisms can and do take many forms, including parliamentary overview committees, courts and law enforcement agencies, elections, citizen charters, investigative journalism and civil society or multi-stakeholder forums (UNDP, 2006).

Recent arguments have posited that the focus of accountability frameworks on answerability, enforcement and sanctions rather than ‘the transformative change of engagement and norms for individuals and institutions’ means that the measurement and success of accountability is often assessed by ‘superficial demonstrations of accountability’ (Martin Hilber *et al.*, 2016) recorded as one-off actions. This may result in a lack of appreciation of the pathway, which accountability mechanisms require to achieve sustained change. However, measuring the efficacy of accountability processes and their durability and effectiveness is challenging as there is a lack of broadly applicable, evidence-based practices and tools for this purpose. The existing literature does permit, however, the identification of a series of ‘accountability markers’, i.e. desirable characteristics of an accountability mechanism that could form the basis of an accountability measurement tool. These markers include (but are not limited to) the existence and functionality of monitoring, review and remedy or action components, multi-stakeholder/multi-sectoral engagement, political will, effective leadership, accessible and good quality monitoring data, supportive champions who can draw attention to performance, the taking of remedial action in response to identified problems or challenges, national ownership and scale to other vertical and horizontal forces, e.g. sub-national ownership, minister-to-minister/governor-to-governor and private-sector counterparts (Fox, 2016).

This article documents an effort to transform accountability markers into a measurement tool that can be used to better assess progress and learning to advance the implementation of health-related accountability interventions. The tool aims to facilitate the assessment of accountability efforts in countries and inform course correction by accountability stakeholders in the field. It can be adapted and used by those implementing an accountability action (to assess progress), or by those who wish to review or evaluate accountability efforts retrospectively. The tool itself and accompanying guidance notes are presented in the Supplementary annex.

The tool was developed and piloted in association with the Evidence for Action (E4A)-MamaYe programme, which is managed by Options Consultancy Services Ltd (Evidence for Action-MamaYe, 2019). A multi-year programme implemented across various countries in sub-Saharan Africa, E4A-MamaYe, supports civil society, government, media and parliamentarians and advocates at global, regional, national and sub-national levels for improved quality of care through improved accountability of decision-makers for prioritization, planning and spending on health.

## Methods

### Literature review

The development of the framework and tool was informed by a structured literature review. In January 2018, we searched PubMed, JSTOR, ScienceDirect, Web of Science and IBSS. The literature search was limited to studies involving human subjects, published in or after 2006 and in English, French or Portuguese. Search terms included ‘accountability’, ‘maternal health’, ‘newborn health’, ‘reproductive health’, ‘child health’, ‘adolescent health’, ‘nutrition’, ‘human rights’, ‘scorecard’, ‘governance’ and ‘death audit’. The search generated 1721 publications, the titles and abstracts of which were screened by one researcher to assess whether or not the paper described or assessed an accountability mechanism. A total of 44 did so and went forward for full-text review. Data were extracted for each paper as follows:

- approach,
- type of mechanism,
- accountability tool(s) used as part of mechanism,
- country/ies and/or region(s) in which the mechanism operated,
- whether the mechanism related to maternal, newborn, child or adolescent health, or to nutrition,
- who was (or should have been) accountable to whom,
- administrative level at which the mechanism operated,
- health system level at which the mechanism operated,
- whether or not there was an attempt to measure accountability,
- stakeholder(s) involved, and
- characteristics of the mechanism that may be relevant to the development of a set of assessment criteria, e.g. enabling factors and barriers to sustainable implementation of the mechanism

The thinking on accountability has evolved from an early focus on governance to more recent efforts to focus on what it aims to achieve answerability for. In the health domain, an early classification divided accountability mechanisms into the following three dimensions: performance accountability, political/democratic accountability (which includes social accountability) and financial accountability (Brinkerhoff, 2001)—a typology now officially used by the World Health Organisation:

- Performance accountability: scrutiny of the actions of officials in relation to the delivery of services, accomplishment of objectives and achievement of results.
- Political accountability: oversight of officials and their responsiveness to citizens.
- Financial accountability: compliance of officials with laws, regulations and procedures for the transparent allocation, expenditure and reporting of financial resources.

Table 1 shows that most of the reviewed papers related to performance accountability, with some literature related to political accountability—specifically articles on social accountability and human rights. However, the more limited search results on political accountability could be a limitation of the search strategy. While many actors and organizations support work that shares many of the attributes of what we call ‘accountability’, they do not label it as such.

**Table 1:** Characteristics of accountability mechanisms described in reviewed papers

	Number of papers (*N* = 44)
Type of mechanism	
Performance accountability	
MDSR or similar	18
Professional organizations	3
Assessment tool or scorecard	4
Political and democratic accountability	
Social accountability	7
Political accountability	0
Human rights	2
Financial accountability	
Financial and budget tracking schemes	7
Performance-based payment schemes	3
Health topic^a^	
Maternal health	37
Newborn/neonatal health	18
Reproductive health	6
Child health	8
Adolescent health	1
Nutrition	0
Location	
Sub-Saharan Africa	33
South Asia	4
Others (e.g. global)	7

MDSR, maternal death surveillance and response.

a Several papers covered more than one topic, so the total is >44.

The vast majority of papers were about maternal and newborn health (MNH), with a few about reproductive, child or adolescent health. Most of the studies took place in Sub-Saharan African countries, with a few from South Asia and a few from global studies. Almost half related to maternal death surveillance and response-type systems.

While searching for relevant published papers, we also identified a number of relevant publications that did not describe an accountability mechanism per se but did provide information or ideas that were considered relevant to the development of an accountability framework and tool. Sixty-seven of these items were reviewed, and information about them recorded in an abridged version of the extraction grid. Of those reviewed, 22 were considered to include information relevant to inform the framework and review under study and therefore fed into the development of the framework and tool. We reviewed information on the websites of relevant organizations and networks that featured heavily in the literature. In addition, we briefly reviewed key grey literature, including the 2017 Independent Accountability Panel (IAP) report (EWEC Independent Accountability Panel, 2017), and documentation relating to the UAF for the Global Strategy for Women’s, Children’s and Adolescents’ Health (EWEC, 2017).

### Development of an accountability framework

It was considered important to align our framework and tool with relevant global architecture, such as the UAF and the IAP accountability framework. This was appropriate because the literature highlighted that the same fundamental process of ‘monitor–review–act’ (i.e. the heart of the IAP framework) applies to the implementation of an accountability mechanism regardless of the type of mechanism or the issue it is attempting to address. However, it was also clear from the literature that there are stages to a successful accountability mechanism that occur prior to the ‘monitor’ stage and stages that occur after the ‘action’ phase. This was encapsulated by Belizán *et al.* (2011), who proposed the following three phases to an accountability mechanism: pre-implementation, implementation and institutionalization. Table 2 shows how the IAP accountability framework of ‘monitor–review–act’ fits into the implementation phase (and may go through more than one iteration). It is assumed that, once those responsible have accepted an accountability mechanism – usually the government, it becomes a routine part of the way in which answerability is assured: this process we call institutionalization, which when sustained can transform processes definitively.

**Table 2:** Phases of an accountability mechanism

Phase			Description
1	Pre-implementation		Awareness raising, commitment from relevant stakeholders, designing the mechanism
2a	Implementation	Monitor	Design and application of data collection instruments, analysis and packaging of monitoring data
2b		Review	Discussion of monitoring data, identification of issues and possible solutions
2c		Remedial action	Changes to policies and/or practices that aim to address the identified issues and problems
3	Institutionalization		Processes and changes are integrated into routine practice and sustained over time through an embedded and functional accountability mechanism

The premise supporting the scheme in Table 2 is that a functional mechanism will complete the ‘implementation’ phase, but an effective one (i.e. one that leads to sustained improvements at the outcome and impact level) must also complete the pre-implementation and institutionalization phases. Most of the mechanisms described in the literature stopped short of institutionalization, and many of the reviewed papers acknowledged this as a shortcoming. However, a few papers described how accountability mechanisms had brought about sustained changes to norms, perceptions and/or practices at the outcome level that in some cases were assumed to have caused changes at the impact level (Rhoda *et al.*, 2014; Bayley *et al.*, 2015; Biswas, 2017). These papers indicated that there should be a distinction between institutionalization (which occurs when there are sustained, country-driven changes to embedded ‘processes’, such as quality improvement activities, staff shift patterns or systems for maintaining stocks of essential medicines) and transformation (which occurs when there are changes to ‘norms and/or policies’). This led us to add a fourth phase to our accountability framework: transformation.

The literature review highlighted a number of desirable characteristics of an accountability mechanism that could be considered as ‘accountability markers’. These are set out in Table 3.

**Table 3:** Accountability markers identified via the literature review

Phase	Accountability markers	Papers identifying this marker
All	Answerability of the duty bearer(s) to the rights holder(s)	Powell-Jackson *et al.* (2006), Kaur (2012), Ray *et al.* (2012), Armstrong *et al.* (2014), Garba and Bandali (2014), Abe *et al.* (2015), Gullo *et al.* (2017) and Schaaf *et al.* (2017)
	Multi-sectoral, multi-stakeholder involvement in the process	South Africa Every Death Counts Writing Group *et al.* (2008), Cottingham *et al.* (2010), Mbeeli *et al.* (2011), Belizán *et al.* (2011), Bellows *et al.* (2013), Armstrong *et al.* (2014), Garba and Bandali (2014), Crofts *et al.* (2014), De Brouwere *et al.* (2014), Abe *et al.* (2015), Bayley *et al.* (2015), Ekirapa-Kiracho *et al.* (2016), Rout *et al.* (2016), Bandali *et al.* (2016), Blake *et al.* (2016), de Kok *et al.* (2017) and Gullo *et al.* (2017)
	Effective leadership	Powell-Jackson *et al.* (2006), Dumont *et al.* (2006; 2009), Nyamtema *et al.* (2010), Cottingham *et al.* (2010), Belizán *et al.* (2011), Armstrong *et al.* (2014), Hofman and Mohammed (2014), Rhoda *et al.* (2014), Garba and Bandali (2014), Arregoces *et al.* (2015), Waiswa *et al.* (2016), Gutschow (2016), Biswas (2017) and de Kok *et al.* (2017)
	Capacity to implement	Hussein *et al.* (2009), Cottingham *et al.* (2010), O’Meara *et al.* (2011), Belizán *et al.* (2011), Bellows *et al.* (2013), Armstrong *et al.* (2014), Rhoda *et al.* (2014), De Brouwere *et al.* (2014), Hofman and Mohammed (2014), Kismodi *et al.* (2015), Moshabela *et al.* (2015), Bandali *et al.* (2016), Moyer *et al.* (2016), Waiswa *et al.* (2016), Wilhelm *et al.* (2016), Biswas (2017) and de Kok *et al.* (2017)
	Independence of the mechanism from the duty bearer(s)	Ray *et al.* (2012) and Garba and Bandali (2014)
	Enabling environment	South Africa Every Death Counts Writing Group *et al.* (2008), Belizán *et al.* (2011), Crofts *et al.* (2014), Rhoda *et al.* (2014), Bandali *et al.* (2016), Wilhelm *et al.* (2016) and Mafuta *et al.* (2017)
1. Pre-implementation	Political will	South Africa Every Death Counts Writing Group *et al.* (2008), Cottingham *et al.* (2010), Nyamtema *et al.* (2010), Mbeeli *et al.* (2011), O’Meara *et al.* (2011), Ray *et al.* (2012), Yilla *et al.* (2014), De Brouwere *et al.* (2014), Garba and Bandali (2014), Bandali *et al.* (2016), Blake *et al.* (2016), Wilhelm *et al.* (2016) and Gutschow (2016)
	Stakeholder commitment (rights holders and/or duty bearers as appropriate)	Mogobe *et al.* (2007), Nyamtema *et al.* (2010), Belizán *et al.* (2011), Mbeeli *et al.* (2011), Ray *et al.* (2012), Armstrong *et al.* (2014), Yilla *et al.* (2014), Crofts *et al.* (2014), De Brouwere *et al.* (2014), Hofman and Mohammed (2014), Bandali *et al.* (2016), Waiswa *et al.* (2016), Gutschow (2016), Schaaf *et al.* (2017) and Gullo *et al.* (2017)
	Partnership structures (to ensure that all stakeholders can participate)	Belizán *et al.* (2011), Ray *et al.* (2012), Garba and Bandali (2014), Ekirapa-Kiracho *et al.* (2016), Waiswa *et al.* (2016), Gullo *et al.* (2017) and Schaaf *et al.* (2017)
	Appropriate design for the context	Hussein *et al.* (2009), Cottingham *et al.* (2010), Belizán *et al.* (2011), Bellows *et al.* (2013), Hounton *et al.* (2013), Crofts *et al.* (2014), Arregoces *et al.* (2015), Waiswa *et al.* (2016), Wilhelm *et al.* (2016), Blake *et al.* (2016), Ekirapa-Kiracho *et al.* (2016), Schaaf *et al.* (2017) and Gullo *et al.* (2017)
2a. Monitor	Data quality and transparency	Powell-Jackson *et al.* (2006), Dumont *et al.* (2006; 2009), Mogobe *et al.* (2007), Hussein *et al.* (2009), Mbeeli *et al.* (2011), Pirkle *et al.* (2013), Sidze *et al.* (2013), Armstrong *et al.* (2014), Rhoda *et al.* (2014), Yilla *et al.* (2014), Crofts *et al.* (2014), Hofman and Mohammed (2014), Arregoces *et al.* (2015), Mathai *et al.* (2015), Bayley *et al.* (2015), Lebbie *et al.* (2016), Rout *et al.* (2016), Bandali *et al.* (2016), Waiswa *et al.* (2016), Wilhelm *et al.* (2016), Gutschow (2016) and Biswas (2017)
	Data presentation	Nyamtema *et al.* (2010), Ray *et al.* (2012), Crofts *et al.* (2014), Garba and Bandali (2014), Yilla *et al.* (2014), Bandali *et al.* (2016), Moyer *et al.* (2016), Waiswa *et al.* (2016) and Rotheram-Borus *et al.* (2017)
2b. Review	Focus on solutions (as opposed to blame)	South Africa Every Death Counts Writing Group *et al.* (2008), Dumont *et al.* (2009), Hussein *et al.* (2009), Ray *et al.* (2012), Armstrong *et al.* (2014), Hofman and Mohammed (2014), Rhoda *et al.* (2014), Bandali *et al.* (2016), Gutschow (2016) and de Kok *et al.* (2017)
	Appropriate solutions that address the identified issues	Armstrong *et al.* (2014), Crofts *et al.* (2014), Hofman and Mohammed (2014), Bandali *et al.* (2016), de Kok *et al.* (2017) and Gullo *et al.* (2017)
	Feedback loop involving both rights holder(s) and duty bearer(s)	Dumont *et al.* (2009), Nyamtema *et al.* (2010), O’Meara *et al.* (2011), Hounton *et al.* (2013), Bellows *et al.* (2013), Armstrong *et al.* (2014), Crofts *et al.* (2014), De Brouwere *et al.* (2014), Bayley *et al.* (2015), Bandali *et al.* (2016), Gutschow (2016), Ekirapa-Kiracho *et al.* (2016), Schaaf *et al.* (2017), de Kok *et al.* (2017) and Gullo *et al.* (2017)
	Equity (giving appropriate consideration to all affected sub-groups)	Powell-Jackson *et al.* (2006), Mbeeli *et al.* (2011), Kaur (2012), Hounton *et al.* (2013), Ekirapa-Kiracho *et al.* (2016) and Gutschow (2016)
2c. Remedial action	Incentives for action	Nyamtema *et al.* (2010), Ray *et al.* (2012), Bellows *et al.* (2013), Rhoda *et al.* (2014), Bandali *et al.* (2016) and Gutschow (2016)
	Consequences for inaction	Nyamtema *et al.* (2010), Ray *et al.* (2012) and Schaaf *et al.* (2017)
	Attribution/contribution (establishing the extent to which the mechanism contributes to action being taken)	O’Meara *et al.* (2011), Hounton *et al.* (2013), Crofts *et al.* (2014), Bandali *et al.* (2016), Lebbie *et al.* (2016), Gullo *et al.* (2017) and Rotheram-Borus *et al.* (2017)
	Feedback loop involving both rights holder(s) and duty bearer(s)	Dumont *et al.* (2009), Issah *et al.* (2011), Hounton *et al.* (2013), Bellows *et al.* (2013), Armstrong *et al.* (2014), Yilla *et al.* (2014), Crofts *et al.* (2014), De Brouwere *et al.* (2014), Bayley *et al.* (2015), Bandali *et al.* (2016), Gutschow (2016), Ekirapa-Kiracho *et al.* (2016), de Kok *et al.* (2017) and Gullo *et al.* (2017)
3. Institutionalization	National (not just local) ownership of the mechanism	South Africa Every Death Counts Writing Group *et al.* (2008), Armstrong *et al.* (2014), Rhoda *et al.* (2014), Yilla *et al.* (2014), Crofts *et al.* (2014), De Brouwere *et al.* (2014), Bayley *et al.* (2015), Mathai *et al.* (2015), Moshabela *et al.* (2015), Bandali *et al.* (2016), Wilhelm *et al.* (2016), Ekirapa-Kiracho *et al.* (2016), Schaaf *et al.* (2017), Gullo *et al.* (2017) and Mafuta *et al.* (2017)
	Sustained change to process(es)	South Africa Every Death Counts Writing Group *et al.* (2008), Crofts *et al.* (2014), De Brouwere *et al.* (2014), Rhoda *et al.* (2014), Yilla *et al.* (2014), Bandali *et al.* (2016), Anselmi (Binyaruka and Borghi (2017), Gullo *et al.* (2017) and Schaaf *et al.* (2017)
	Attribution/contribution (establishing the extent to which the mechanism contributes to the identified changes to processes)	Issah *et al.* (2011), O’Meara *et al.* (2011), Hounton *et al.* (2013), Armstrong *et al.* (2014), Crofts *et al.* (2014), Garba and Bandali (2014), Abe *et al.* (2015), Lebbie *et al.* (2016), Anselmi (Binyaruka and Borghi (2017) and Gullo *et al.* (2017)
4. Transformation	National (not just local) ownership of the mechanism	South Africa Every Death Counts Writing Group *et al.* (2008), De Brouwere *et al.* (2014), Abe *et al.* (2015), Bayley *et al.* (2015), Mathai *et al.* (2015), Moshabela *et al.* (2015) and Gullo *et al.* (2017)
	Sustained change to norms/policies	Ray *et al.* (2012), Abe *et al.* (2015), Bandali *et al.* (2016), Gutschow (2016), Gullo *et al.* (2017) and Schaaf *et al.* (2017)
	Contribution (establishing the extent to which the mechanism contributes to the identified changes to norms/policies)	O’Meara *et al.* (2011), Hounton *et al.* (2013), Armstrong *et al.* (2014), Abe *et al.* (2015) and Anselmi *et al.* (2017)

**Figure 1:**
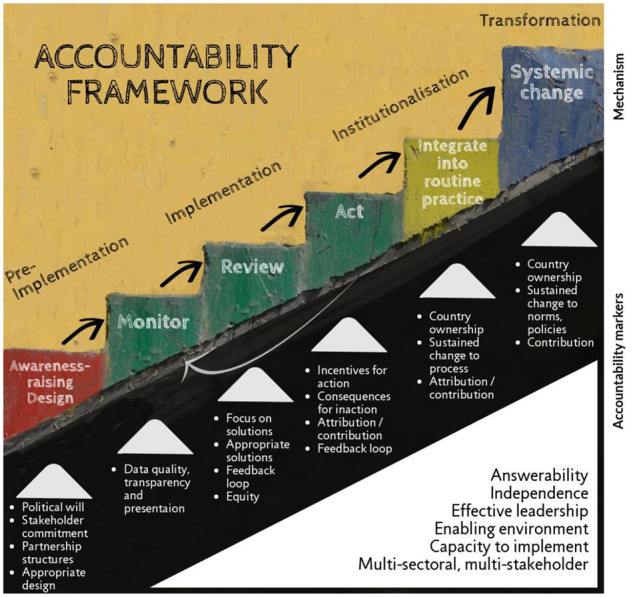
Accountability framework

Although the literature demonstrated that often it is necessary to go through the ‘monitor–review–act’ loop more than once, the four phases generally occurred in chronological order, i.e. it is necessary to complete the earlier phases before addressing the later ones. The fact that relatively few papers described mechanisms that reached phases 2c, 3 or 4 implies that it is more difficult to progress to these later stages, especially the transformation stage. The framework in Figure 1 illustrates these characteristics by including an arrow going back from ‘act’ to ‘monitor’ before proceeding to institutionalization and depicting the different phases as a set of ascending stairs, with the height of the final step being greater than the height of the earlier ones as it is more difficult to achieve.

### Development of an accountability measurement tool

To maximize accessibility to teams working on the implementation of accountability mechanisms, our aim was to develop a tool that that could be completed by those directly involved in the implementation of accountability processes. As mentioned previously, the tool was designed to be adaptable and applied flexibly, including as a baseline tool to inform where there are gaps in the processes, to monitor established accountability mechanisms and provide an opportunity for self-reflection or to retrospectively explore impact where a mechanism has been operational for long enough to have made a difference to accountability. It can also be adapted for other thematic areas beyond health. A summary of the tool is provided in Table 4, and the tool itself can be found in Supplementary Annex A.

**Table 4:** Summary components of the tool

Section	Marker
A. Context	
B. Stakeholder analysis	
C. Pre-implementation phase	C1. Multi-sectoral, multi-stakeholder
	C2. Political will and stakeholder commitment
	C3. Appropriate design
D. Implementation phase	D1. Effective leadership and management
	D2. High quality monitoring data
	D3. Solution-focused review of data
	D4. Remedial action in response to review
E. Institutionalization	E1. Contribution to sustained change
	E2. Country ownership
	E3. Evaluation and scale-up
F. Transformation	F1. Contribution to systematic change

The tool includes a brief explanation of what it is designed to do, and how it should be used, stressing the importance of seeking the input of a wide range of stakeholders when completing the tool, rather than relying on the opinions and experiences of a few. The tool comprises six sections (Table 4). Section A requests contextual information about the mechanism, e.g. the type of mechanism, where it operates and its objectives. Section B is a stakeholder analysis, to record which stakeholders were involved in the mechanism and in what capacities. Sections C–F assess progress through the phases of the framework, from monitoring actions to transforming processes to be more accountable.

In Figure 1, the accountability markers that need to happen or be in place for that phase to be successful are shown underneath the relevant process step(s). Each accountability marker listed in Table 3 is assessed using one or more criteria. For each criterion, the assessor allocates a score between 3 (criterion is fully met) and 0 (criterion is not even partly met) or indicates that the criterion is not relevant to that particular mechanism. There is space to record notes and supporting evidence, e.g. explanation as to why the criterion is not relevant. An accompanying guidance note (Supplementary Annex B) explains in detail what ought to be in place for a particular score to be allocated.

The tool has been designed in order that participants can self-administer it once they have built up familiarity and confidence with the approach and value of the tool and its assessment criteria. For this reason, during the fieldwork pilot, we used an independent consultant to ensure that a discussion around the purpose and framing of the tool was facilitated and feedback on improvements and relevance of the tool for that mechanism were captured.

## Expert review and field test

In August 2018, we conducted a pilot test of the tool, comprising the following two phases: expert review and field test. The two phases were conducted separately but concurrently so did not influence each other and used the same version of the tool.

### Expert review

#### Method

Five experts [academics, non-governmental organizations and national-level accountability mechanism implementers] were individually briefed on the background, purpose and objective of the tool and then guided through the tool and accompanying guidance notes. Each reviewer provided their feedback individually, without discussion between reviewers.

#### Results

Feedback from the expert panel (Supplementary Annex C) emphasized the need for the tool to be adapted to context, rather than a one-size-fits-all approach. For example, depending on the length of time, the mechanism has been operational, some questions around decisions taken in the pre-implementation stage (including, whether identified stakeholders were engaged in the design process before the mechanism began to be implemented) may not be appropriate or yield useful information. In this instance, panellists felt that other questions related to political will and commitment and the existence of a clear strategy for the mechanism that has (ongoing) approval by all stakeholders would be more appropriate.

Relatedly, some respondents questioned the relative perspectives and validity of summary scores derived from the scores for individual criteria. In addition, several questions were queried on the basis that they were more relevant for some stakeholders such as programme implementers than for others (e.g. funders)—e.g. questions around scale-up of the mechanism model to a larger geographic area. For long-established mechanisms, this question may have varied relevance or not apply to stakeholders within the mechanism but rather be more relevant to national structures and/or programme implementers. Where this is the case, it inevitable raises questions about how accountability initiatives are fully embedded/sustainable within sub-national and national contexts—both larger questions the tool seeks to motivate stakeholders to reflect on.

Part of the consultation involved asking for views on how the tool could be scored and whether specific criteria should carry more weight than others in an overall assessment of the effectiveness of the mechanism. While there was harmony on the importance of some questions—e.g. the need for clear objectives and plans, the need for champions and/or influential leaders, the need for the review process to propose solutions—there was variation in the weight different individuals placed on certain markers. Considering this alongside the other points raised in the consultation and pilot (discussed shortly), we believe that the scoring is likely to be unique to the mechanism itself and it is not necessarily desirable to compare mechanisms based on aggregate scoring.

Finally, there was agreement among all consulted on the need for a greater focus on the actions taken in response to review and whether these contributed to any changes and the evidence for this result.

### Field test

#### Method

A core criterion for the field test was that the accountability mechanisms were well-established, enabling all sections of the tool to be properly tested. The test involved two different accountability mechanisms selected as they represent different types of accountability in Nigeria: (1) The Bauchi State Accountability Mechanism for Maternal, Newborn and Child Health [a State Led Accountability Mechanism (SLAM)], which is a social accountability mechanism, and (2) the Gombe State Maternal and Perinatal Death Surveillance and Response (MPDSR) Steering Committee, which is a performance accountability mechanism.

SLAMs are multi-stakeholder mechanisms, with membership from government bodies, civil society, health professional associations and the media. SLAMs have two co-chairs, one representing the Ministry of Health and one representing civil society. The mandate of the SLAM is to review and monitor progress on key MNCH indicators (decided by the SLAM) and co-jointly develop solutions and actions.

MPDSR Steering Committees exist at national, state and facility level in Nigeria. Structure and membership follow the National Guidelines on MPDSR Committees.

In Gombe, membership comprises State Ministry of Health officials, members of the Primary Health Care Development Board and civil society. Quarterly review meetings are held and progress across the State's secondary health facilities is reviewed and included into a State MPDSR Scorecard, which includes indicators and calls to action.

Although the tool is designed to be self-administered by those implementing the accountability mechanism, the field test was facilitated by an external consultant based in Nigeria. This enabled the provision of a detailed pilot report based on both the feedback of the participants and the informed observations of the facilitator.

For each mechanism, the facilitator attended a meeting with stakeholders to facilitate a discussion and test the tool. All participants were briefed on the background, objective and purpose of the tool and asked for their consent for the results of the discussion to be recorded. In Gombe, the meeting involved 33 members of the MPDSR State Steering Committee, including representatives from the State Ministry of Health, the State Primary Health Care Development Agency, hospitals and Health Management Information System department. In Bauchi, the meeting involved 32 representatives, including civil society organizations and government representatives. It should be noted that neither meeting achieved participation from all stakeholders: something that is also common to regular meetings where full attendance is not always guaranteed.

Participants were guided through the questions and scoring criteria and were encouraged to achieve consensus on scores for each criterion. If a consensus could not be reached, reasons for the disagreement and the desired scores of each party were noted. At the end of the discussion, participants were asked questions about what they thought of the process of applying the tool, the scoring and whether they would take any action as a result of their discussions.

#### Results

Both mechanisms scored highly on the pre-implementation phase, which was perhaps to be expected given that both mechanisms are well-established. As the assessment moved into the implementation, institutionalization and transformation phases, the scores become more mixed (Table 5). Despite the expressed satisfaction of participants with the accountability efforts of the mechanisms, they felt that some of their achievements were not well captured by the tool because the mechanisms did not always reliably document their own activities and results.

**Table 5:** Summary of pilot scores

Section	Bauchi SLAM	Gombe MPDSR State Committee
A—Context	–	–
B—Stakeholder analysis	–	–
C—Pre-implementation phase	24/27 (89%)	24/27 (89%)
D—Implementation phase	49/81^a^ (60%)	55/87^a^ (63%)
E—Institutionalization	9/21^a^ (43%)	6/21^a^ (29%)
F—Transformation	6/12 (50%)	6/12 (50%)
Total	88/141 (62%)	91/147 (62%)

a The denominator excludes not applicables (individual criteria that were judged to not be applicable to the mechanism).

As with the expert panel, interpretation of some of the definitions and scoring procedures differed and needed clarification. Rigid definition of assessment criteria and assumptions of prior knowledge of the literature on accountability and/or an awareness of how decisions were made during inception [e.g. evaluation and scale-up marker (E3)] made some of the questions difficult to answer for one or both mechanisms. For a complete and triangulated assessment of the mechanisms, it will be necessary to conduct the exercise with both programme managers/funders and participants in the mechanism, something that was not possible during the pilot.

In the pilot version of the tool, the appropriate design marker (C3) had three assessment criteria, rather than the current two. The third criterion aimed to ascertain if there was ‘evidence that, if implemented well, this type of mechanism can address the problem(s)/issue(s) it was designed to address’. Pilot participants did not feel qualified to comment about evidence from other settings and so did not feel able to answer. In addition, both pilot participants and expert reviewers felt that it is equally important to innovate and try different approaches as it is to implement practices and programmes that have proof of impact. The decision was therefore taken to delete this criterion.

There were instances when pilot participants responded ‘not applicable’ when the mechanism should have been scored a ‘0’ for its performance against that criterion. For example, on the evaluation and scale-up marker (E3), both mechanisms wrote NA against all criteria, whereas a score of 0 would have captured more accurately what both mechanisms noted—that this was the first evaluation of the mechanism. This may indicate a lack of a shared understanding by participants on how the scoring criteria in these examples were to be used and perhaps not enough discussion on the difference between NA and 0. However, it may also indicate a reluctance to give a score of 0 to avoid the appearance of having ‘failed’ on one or more of the markers. To help avoid this problem, more detailed instructions were added to the tool, referring users to the guidance notes if they are considering a response of ‘NA’.

For both mechanisms, participants remarked that the exercise highlighted areas of their work that needed specific attention. In particular, generating evidence of whether and how the accountability mechanisms contribute to the transformation of health indicators, and where they could strengthen their own efforts within the accountability cycle to improve these outcomes. It was also useful at highlighting areas of disagreement among participants and generating a discussion on different perspectives and what needed to be done to reconcile these practically through the future work of the mechanism.

An overall score was calculated based on a combination of the scores for the individual criteria, as shown in Table 5. However, the field test indicated that the process of applying the tool was in itself sufficient to evaluate the effectiveness of the mechanism and pinpoint areas that required strengthening. If the tool is being used for this purpose, an overall score might not be necessary. If it is being considered for use as a monitoring tool, an overall score that can be tracked over time might be desirable. In that case, careful consideration will need to be given to the relative weight applied to each accountability marker and/or assessment criterion. Who is involved in this process will need to be considered from the perspective of what subsequent validity the scores need to have and for whom.

Participants in each accountability mechanism felt that the tool took too long to complete (4 h each). Suggestions to improve the process include spreading out the discussion over a couple of days and prioritizing questions for specific audiences to reduce the time it takes to implement the tool without losing the value of the exercise.

## Discussion

Efforts to better understand and measure the effectiveness of accountability interventions to improve health outcomes has received considerable attention in the last few years (Holland and Schatz, 2016; USAID, 2016; COPASAH, 2019; CORE Group, 2019a; GPSA, 2019; Nove *et al.*, 2019; White Ribbon Alliance, 2019). Accountability is recognized as central to applying human rights to development and health, and social accountability in particular has been acknowledged as a crucial element of the enabling environment for achieving UHC and quality of health care. Recent conventions, reviews and articles have moved the conversation forward by seeking to understand not only the nature of interventions and their effectiveness but also the context, actors and processes by which greater accountability can be built. There has been a particular interest in how to measure and evaluate the effectiveness of accountability efforts (Tolmie and Arkedis, 2015; GPSA, 2018). Randomized controlled trials (RCTs) have provided mixed results, which has led to a push for more nuanced and contextual evaluations (which can accommodate complexity) to build the evidence base on accountability for improved outcomes (Westhorp *et al.*, 2014). We know of no tools currently being used, however, to self-assess progress towards accountability.

The need for contextualization of actions and contributions is supported by a recent review highlighting how accountability mechanisms operate within a ‘complex “accountability ecosystem”’ (Van Belle *et al.*, 2018). In this ecosystem, accountability actors such as those running the SLAM do so within a web of interactions, roles and responsibilities that affect levels of engagement and often, the relative success of the efforts. This context in fact may underscore whether an accountability action will be successful or not and must also be taken into consideration.

There are few examples in the peer-reviewed literature of interventions aimed at strengthening accountability mechanisms that lead to institutionalized change and transformation of norms and practices (Nove *et al.*, 2019). Results of the field test demonstrate that despite wide appreciation and regard for accountability interventions, mechanisms tend not to document evidence of their effectiveness in bringing about sustained change. For example, in the case of the SLAM and MPDSR Steering Committee, there was a perception among those involved in completing the tool that the overall scores did not fully reflect the achievements of the mechanism: while engagement of actors was robust, documentation of the effect of the efforts limited the extent to which results could be demonstrated. A recent review of performance accountability (which used an early version of the accountability framework presented here) similarly highlighted a lack of documentation of how greater accountability and sustained changes in the health system were achieved via monitoring, review and remedial action for the work of the mechanism itself (Nove *et al.*, 2019) This suggests a need for accountability mechanisms to incorporate a more thorough and ongoing review and documentation of their actions and contributions, and not just a review of health system data. This review could include, e.g. tracking their own action plans, how these were implemented and to what quality standard. In doing so, mechanisms will be better able to hold themselves accountable for their progress in contributing to achievements in health outcomes. However, even in the absence of such documentation, a tool such as the one presented in this paper can be a useful exercise to assess what is working and identify areas where greater investment is needed.

The accountability framework presented here attempts to provide actors engaged in strengthening accountability mechanisms with a practical tool to assess progress in context. As a self-reflection tool, implementers of accountability interventions are guided through an assessment of their efforts and the extent to which they adhere to established good practice in accountability. The process asks participants to consider whether the approaches employed have the necessary stakeholder engagement, information and analysis to define and push remedial action that can be implemented and sustained, thereby increasing accountability of the system. A consensus-based scoring process allows stakeholders to identify the strengths and weakness of their approach and better understand why progress was made or stalled and where course correction is needed. The tool can be used retrospectively or prospectively to reflect or plan accountability interventions and identify areas for improvement. It was designed to assess accountability mechanisms related to MNH but can be applied broadly to the field global health and beyond as the steps are focused on process rather than thematic content.

Presently, the tool is being further adapted and tested as a framework for analysing case studies related to the institutionalization of accountability mechanisms.[Bibr czz170-B8] Results of the case studies will be published later in 2020.

## Conclusion

It is widely recognized that progress on global goals related to UHC and the Global Strategy for Women’s, Children’s and Adolescents’ Health will require greater commitment and accountability at all levels. Those with an obligation or responsibility to provide as well as those entitled to those provisions/or advocating for them are implicated together as accountability stakeholders, as both are required to achieve the institutional changes needed to improve health systems, services and ultimately health outcomes. There is a need to broaden and develop how we plan, monitor and evaluate accountability, particularly social accountability, beyond RCTs on the one hand and qualitative approaches on the other hand. Recent examples of realist reviews are a step forward. Implementers could be further aided by new tools to measure progress and document how and why some approaches are successful while others fail to gain traction. The accountability framework presented here is offered as a contribution towards our collective effort to understand, contextualize and ultimately increase accountability for improved health outcomes.

## Supplementary data

Supplementary data are available at *Health Policy and Planning* online.

## Supplementary Material

czz170_supplementary_data
