# Supplementary material for: The development of a new accountability measurement framework and tool for global health initiatives
Source: Health Policy Plan. 2020 Jun 3;35(7):765–74. doi: 10.1093/heapol/czz170 (PMC7487333; doi:10.1093/heapol/czz170)
Supplement: czz170_supplementary_data [file czz170_supplementary_data.zip › Annex A_The Tool.docx]

# Annex A: The tool

**Accountability Tool: measurement questionnaire**

The purpose of this tool is to facilitate measurement of accountability processes by implementers. It aims to allow those who engage in accountability mechanisms to conduct a self-reflection exercise to assess the potential efficacy of their efforts. By working through the tool, accountability stakeholders can assess the results they contribute to, how and why their actions are working to increase accountability towards health-related outcomes, as well as what may need strengthening to show better results. Designed as a learning tool it seeks to improve efforts to increase accountability in the health sector.

The tool is structured against four phases of implementation, each with several accountability markers. The four phases are:

Pre-implementation

Implementation (including, monitor, review, remedial action)

Institutionalisation

Transformation

This tool is best used as an evaluation tool by stakeholders working to strengthen accountability mechanisms. It can also be adapted to be used prospectively to help design approaches. Users of the tool should consider and adapt the terminology within the tool to reflect their own context: for example, the terms rights-holders and duty-bearers will need to be replaced within the tool to reflect what these posts mean / refer to within the context – e.g., service user / service provider.

The tool has been designed to be self-administered so that accountability actors can convene a meeting to go through the tool as a group and assign scores against the relevant criterion related to their own efforts to strengthen an accountability mechanism. Users can also hire an external facilitator to guide and moderate this process if they so wish. The accompanying excel guidance sheet provides clarity on the scoring criteria with examples of what might constitute a 0,1,2,3 against each criterion. Reference to the criterion should be made throughout the exercise. Finally, we would encourage all users to have a discussion of the scores following the exercise, focusing specifically on what this means for how they structure and organise their activities in the future.

**Section A: Context**

Please answer the following questions in relation to the accountability mechanism being assessed. Use the [glossary](#_Glossary) for definitions, though terminology may need to be adapted to suit / be appropriate to the context.

| **Q. No.** | **Question** | **Response** |
| --- | --- | --- |
| A1 | Name(s) of person(s) who facilitated the completion of this questionnaire |  |
| A2 | Date on which this questionnaire was fully completed (DD/MM/YYYY format) |  |
| A3 | What is the name of the accountability mechanism being assessed? |  |
| A4 | What type of accountability mechanism is it? (See [glossary](#_Glossary).) Select one only. | Performance  Political/democratic  Social  Financial  Other (specify) |
| A5 | Who supported the initiation of this this mechanism and when? (Enter month and year) |  |
| A6 | At what administrative level(s) does the mechanism operate? Select all that apply. | National  State (if federal country)  Province, region, county or similar  District, woreda or similar  Community, village or similar  Other (specify) |
| A7 | Please list the geographical area(s) in which the mechanism operates |  |
| A8 | What problem does the mechanism try to solve, or what situation does it try to improve? |  |
| A9 | What are the specific aims/objectives/asks of the mechanism? |  |
| A10 | What tools and/or guidelines were used during implementation of the mechanism? (e.g. national or local guidelines for the implementation of this type of mechanism, scorecard tools, verbal autopsies) |  |

**Section B: Stakeholder analysis**

In the table overleaf, list all stakeholders who were involved with any stage of the mechanism. If the stakeholder is an individual, state their position as well as their name, e.g. ‘traditional leader’, ‘first lady’ If the stakeholder is an organisation, enter the name of the organisation. For each, state their area of influence (opinion leader/influencer, planning, finance, policy/regulation, implementer of the action, beneficiary - see [glossary](#_Glossary) for definitions that can be adapted for the context), their position in the accountability process (rights holder, duty bearer, implementer, funder – see [glossary](#_Glossary) for definitions that can be adapted to for the context) and indicate whether or not they were involved in the process of completing this questionnaire. Add more rows if needed.

| **Name of stakeholder (position or organisation)** | **Area of influence.**  **Select all that apply** | **Position in the accountability process. Select all that apply** | **Involved in this questionnaire?** |
| --- | --- | --- | --- |
|  | Opinion leader/influencer  Planning  Finance  Policy and regulation  Implementation of the action (manager/provider)  Beneficiary  Other | Rights-holder  Duty-bearer  Implementer  Funder  Other | Yes  No |
|  | Opinion leader/influencer  Planning  Finance  Policy and regulation  Implementation of the action (manager/provider)  Beneficiary  Other | Rights-holder  Duty-bearer  Implementer  Funder  Other | Yes  No |
|  | Opinion leader/influencer  Planning  Finance  Policy and regulation  Implementation of the action (manager/provider)  Beneficiary  Other | Rights-holder  Duty-bearer  Implementer  Funder  Other | Yes  No |
|  | Opinion leader/influencer  Planning  Finance  Policy and regulation  Implementation of the action (manager/provider)  Beneficiary  Other | Rights-holder  Duty-bearer  Implementer  Funder  Other | Yes  No |

To summarise, please complete the following table to indicate the individual(s) and/or organisation(s) who are the duty-bearers and rights-holders under this accountability mechanism (see [glossary](#_Glossary) for definitions). **Both boxes must be completed**: an accountability mechanism always has both duty bearer(s) and rights-holder(s).

| **Role** | **Name(s of individual(s) or organisation(s)** |
| --- | --- |
| Duty-bearer(s): |  |
| Rights-holder(s): |  |

**Section C: Pre-implementation phase**

The pre-implementation phase includes awareness-raising among and securing the commitment of relevant stakeholder groups, and designing the mechanism. A high score in this section indicates a well-designed accountability mechanism with local ownership and the potential to be sustainable and achieve the desired change.

In sections C through to F, award a score for each assessment criterion as follows:

3 = criterion is fully met
2 = criterion is mostly met
1 = criterion is partly met
0 = criterion is not met
NA = criterion not relevant

Please refer to the guidance notes to help you decide on the score for each criterion, **especially if you are thinking of entering ‘NA’ rather than a numeric score. NA should be entered only if the description given in the ‘NA’ column applies to the mechanism. Otherwise, a score between 0 and 3 should always be given. Remember that a low score doesn’t necessarily indicate a problem or a failing – it may simply be that it is too early for that criterion to have been met**. Enter the score in the column headed ‘score’. In the ‘notes and supporting evidence’ column, provide information to help interpret the score, e.g. if a low score has been given, what specifically is not in place? Also provide information about where supporting evidence can be found.

| **Marker** | **Assessment criterion** | **Score** | **Notes and supporting evidence** |
| --- | --- | --- | --- |
| C1. Appropriate type of mechanism | 1. The mechanism has a documented plan, protocol or strategy with clear explanation of the objectives, methods and tools to be applied, and approved by all stakeholders |  |  |
|  | b. The mechanism’s structure and membership is able to influence (e.g. has the power to change or alter) decision-making mechanisms at the level at which it operates (e.g. community, sub-national, state, national) |  |  |
| C2. Political will and stakeholder commitment | a. The mechanism supports a specific global, regional, national or sub-national policy or strategy. [If score >0, state which policy/strategy in the right-hand column] |  |  |
|  | b. National, state/regional or local government (as appropriate) made a formal commitment to establish or improve accountability as evidenced by a publicly available document (plan, strategy, press release, statement) or declaration. |  |  |
|  | c. All non-governmental duty-bearers (see [glossary](#_Glossary)) - if any - made a formal commitment to establish or improve accountability within the relevant system(s) as evidence by a publicly available document (plan, strategy, press release, statement) or declaration. |  |  |
| C3. Multi-sectoral, multi-stakeholder | a. The stakeholders in Section B represent all relevant stakeholder groups and sectors needed to give the mechanism the ability to influence change, e.g. government ministries (health, education, finance, transport), traditional leaders, civil society, communities, private sector, health workers, health service management, media, funder(s), development partners, academics/ experts |  |  |
|  | 1. The stakeholders in Section B represent all relevant stakeholder groups and sectors affected by the mechanism, e.g., government ministries (health, education, finance, transport), traditional leaders, civil society, communities private sector, health workers, health service management, media, funder(s), development partners, academics/ experts |  |  |
|  | c. All stakeholders listed in Section B were identified and engaged in the process before the mechanism began to be implemented |  |  |
|  | d. Structures for interaction between all relevant stakeholders exist, e.g. a committee or forum involving all stakeholders that meets regularly (whether or not they were developed specifically for this mechanism) |  |  |

**Section D: Implementation phase**

The implementation phase comprises three stages: monitor, review and action (including actions that stimulate a response) in response to the review. Usually there is an iterative process of repeating the three stages until changes start to be integrated into routine practice. Cutting across all three of these stages is the need for effective leadership and management of the process. A high score in this section indicates a well-functioning accountability mechanism that has improved accountability. Users of the tool should consider making some of the questions – especially those around the review and act processes – time bound, for example asking stakeholders to consider these questions with reference to the last year. In all cases, examples should be asked for.

| **Marker** | **Assessment criterion** | **Score** | **Details and supporting evidence** |
| --- | --- | --- | --- |
| D1. Effective leadership and management | a. Relevant leader(s) and/or champion(s) (see [glossary](#_Glossary)) drove the process of implementation of the mechanism |  |  |
|  | b. The necessary funding, equipment and skilled human resources were allocated to implement the mechanism to its specification |  |  |
|  | c. All duty-bearers are answerable to the rights holders (see [glossary](#_Glossary)), i.e. obliged to provide information about their decisions and actions, and to justify them |  |  |
|  | d. The mechanism operates with a philosophy of learning and improving rather than blaming and shaming/punitive action |  |  |
|  | e. All duty-bearers (see [glossary](#_Glossary)) are aware of their role in the mechanism and what is expected of them |  |  |
|  | f. All duty-bearers (see [glossary](#_Glossary)) carried out their allocated role in the mechanism to a high standard |  |  |
|  | g. The operation of the mechanism is not unduly influenced by politics and vested interests |  |  |
| D2. High quality monitoring data | a. The mechanism involves the collection of monitoring data to track the extent to which the objectives of the mechanism as described at question A9 are being achieved [If score >0, please describe the type of data yielded by the mechanism] |  |  |
|  | b. Monitoring data are complete (i.e. all relevant data items are collected) |  |  |
|  | c. Monitoring data have been rigorously assessed and found to be accurate |  |  |
|  | d. Monitoring data are collected on a regular or ongoing basis so they can be reviewed while still recent and relevant |  |  |
|  | e. Monitoring data are packaged and presented for review in a way that is easily understood by all stakeholders |  |  |
| D3. Solution-focused review of data | a. The mechanism includes a formal process of reviewing the monitoring data |  |  |
|  | b. All stakeholders listed in Section B are represented in the review process |  |  |
|  | c. The review process is equitable, i.e. equal consideration is given to all affected population groups (e.g. women, adolescents, children, disabled people, poor people, minority ethnic groups) |  |  |
|  | d. The review process encourages stakeholders to propose solution(s) to the identified problem(s)/ issue(s) rather than just talking about the problem(s)/ issue(s) |  |  |
|  | e. The proposed solutions take a multi-sectoral, health system approach rather than focusing on a single sector or individual elements of the health system [health system elements include: service delivery, health workforce, health information, medical products/ vaccines/ technologies, health financing, leadership/ governance] |  |  |
|  | f. The proposed solutions are targeted to those with the power to make the necessary change(s) |  |  |
|  | g. There is evidence that the proposed solution(s) have the potential to address the problem(s)/ issue(s) noted at Question A8 |  |  |
|  | h. Learning points and decisions taken as a result of the review were communicated clearly to all relevant internal and external stakeholders |  |  |
|  | i. Relevant stakeholders and/or champions are engaged to help drive and/or implement the specified solution(s) |  |  |
|  | j. The review process yielded documented action plan(s), with clear lines of responsibility |  |  |
| D4. Actions in response to review | a. There are positive incentives for duty-bearers to implement the parts of the action plan that are their responsibility (e.g. additional funding, improved career prospects, public recognition) |  |  |
|  | b. There are consequences for duty bearers who do not implement the parts of the action plan that are their responsibility (e.g., disciplinary procedures, litigation) |  |  |
|  | c. A recourse mechanism (see [glossary](#_Glossary)) was used during implementation of the accountability mechanism, and this brought about positive results [enter ‘NA’ if there was no need to use a recourse mechanism] |  |  |
|  | d. The action plan has been fully implemented |  |  |
|  | e. The actions that were taken were clearly communicated to all relevant stakeholders |  |  |
|  | f. After actions are taken, the ‘monitor and review’ phases are repeated to assess whether the actions have fully addressed the identified problem(s)/ issue(s) |  |  |
|  | g. There have been positive changes to processes that have addressed the problem(s)/ issue(s) noted at Question A8 |  |  |
|  | h. There is evidence that these process improvements took place wholly or partly in response to the review phase of the accountability mechanism [enter NA if score at D4h = 0] |  |  |

**Section E: Institutionalisation**

The institutionalisation phase occurs when an accountability mechanism results in changes to professional norms, standards or functional processes (such as the way staffing protocols are implemented and followed up) that are integrated into routine practice and sustained over a period of time. A high score in this section indicates an effective accountability mechanism that will continue to ensure accountability in the future.

| **Marker** | **Assessment criterion** | **Score** | **Details and supporting evidence** |
| --- | --- | --- | --- |
| E1. Contribution to sustained change | a. Improvements to processes (i.e. those contributing to the score at D4h) have been integrated into routine practice and sustained over a period of time |  |  |
|  | b. There is evidence that the accountability mechanism contributed to the integration of these improvements into routine practice [enter NA if score at E1a = 0] |  |  |
| E2. Country ownership | a. A public sector body or organisation has official responsibility for ensuring that improvements to processes are maintained |  |  |
|  | b. There are positive incentives for this public sector body or organisation to deliver on their responsibility to maintain improvements (e.g. additional funding, public recognition) |  |  |
|  | c. Domestic funds/resources/structures have been made available to help this body or organisation to maintain improvements |  |  |
| E3. Evaluation and scale-up | a. There has been an evaluation of the accountability mechanism, which demonstrated that it had contributed to improved processes or outcomes |  |  |
|  | b. After successful implementation of the mechanism in its initial location(s), it was scaled up across a larger geographical area [If the mechanism is only applicable in one location, or operated at national or state level from the outset, enter ‘NA’] |  |  |
|  | c. After successful implementation of the mechanism in its initial location(s), it was scaled up across the whole country or state [If the mechanism is only applicable in one location, or operated at national or state level from the outset, enter ‘NA’] |  |  |

**Section F: Transformation**

The transformation phase occurs when an accountability mechanism results in systemic changes as well as changes in processes and practices. **Because it is extremely difficult to attribute this kind of systemic change to an accountability mechanism, scores allocated in this section do not count towards the overall score. They do, however, provide an opportunity to identify and showcase mechanisms that have been particularly successful**.

| **Marker** | **Assessment criterion** | **Score** | **Details and supporting evidence** |
| --- | --- | --- | --- |
| F1. Contribution to systemic change | a. There have been changes to behaviour, norms or policies that have addressed the problem(s)/ issue(s) noted at Question A8. Please provide examples. |  |  |
|  | b. There is evidence that the accountability mechanism contributed to these systemic changes. Please provide evidence. |  |  |
|  | c. There have been improvements in health or social outcomes in the locations where the mechanism was implemented. Please provide examples. |  |  |
|  | d. There is evidence that the accountability mechanism contributed to these improved health or social outcomes. Please provide evidence. |  |  |

# **Glossary**

Accountability = when an individual or body, and the performance of tasks or functions by that individual or body, are subject to another’s oversight, direction or request that they provide information or justification for their actions.

Accountability mechanism = a process that is designed to help ensure that those with responsibility for making improvements are held to account for progress (or lack of it) and achieving outcomes.

Beneficiary = an individual or organisation that benefits from or uses the goods or services being provided by a duty-bearer.

Champion = an individual or organisation with social, political or financial power and who uses that power to help ensure that the mechanism is properly implemented.

Duty bearer = an individual or organisation with responsibility for providing goods/services or improving them. Within an accountability mechanism, the duty-bearer is the organisation or individual who is being held to account. Note that a duty-bearer may also be a rights-holder, e.g. a health worker bears the duty to provide health care, and also holds the right to expect their employer to provide an enabling work environment.

Financial accountability = ensuring compliance with commitments, laws, rules and regulations regarding the control and management of financial resources. Examples include budget tracking schemes and performance-based payment schemes.

Influencer = a person or institution that has the social, political or financial power or standing to change public opinion and/or influence policy and practice.

Implementer = an individual or organisation that plays a role in ensuring that the accountability mechanism is functioning.

Monitoring = the use of tools for the systematic collection and presentation of quantitative or qualitative data and evidence, e.g. verbal autopsy forms or scorecards.

Performance accountability = demonstrating and accounting for performance in the light of agreed-upon performance targets, with a focus on services, outputs and results. Examples include maternal death surveillance and response (MDSR) systems, accreditation systems, and annual performance review systems.

Opinion leader – see ‘influencer’.

Political and democratic accountability = use of social, political and legal pressure and judicial processes to pressure, punish or shame institutions or persons responsible such as elected officials, managers, ministers or Ministries by focusing on governance, citizen participation, equity, transparency and openness, responsiveness, and trust. Examples include social accountability campaigns or commitment tracking and assessments, and human rights monitoring and legal action.

Recourse mechanism = a punitive or disciplinary action that is invoked when a duty bearer (individual or institution that is responsible for the action) fails to meet the expectations or criteria established in the improvement process. Examples include transgressions of codes of conduct that are met with professional disciplinary action such as suspension from duties, financial penalties, loss of accreditation.

Remedial action = action taken in response to the monitor and review phases of an accountability mechanism.

Review = the process of drawing conclusions from monitoring data about the strengths and weaknesses of the duty-bearer, and formulating an action plan to address the weaknesses.

Rights holder = a beneficiary with the right to expect the duty-bearer to be held to account for the proper performance of their duties. For example, all individuals have the right to health and therefore the right to expect the health service (the duty bearer) to provide the health care they need, all health workers have the right to a safe workplace and therefore the right to expect their employer (the duty-bearer) to provide this.

Social accountability = a type of political and democratic accountability that engages citizens (rights holders) in accountability processes. Examples include tracking of government commitments in MNH, social audits and complaint mechanisms, petitions, campaigns and protests, and quality of services assessments (scorecards) with community participation.
