# Supplementary material for: The development of a new accountability measurement framework and tool for global health initiatives
Source: Health Policy Plan. 2020 Jun 3;35(7):765–74. doi: 10.1093/heapol/czz170 (PMC7487333; doi:10.1093/heapol/czz170)
Supplement: czz170_supplementary_data [file czz170_supplementary_data.zip › Annex C ExpertReview_Table.docx]

# Annex C: Detailed results from the expert review resultant changes

| **Marker** | **Assessment criterion (original wording)** | **Expert feedback** | **Resultant change** |
| --- | --- | --- | --- |
| C1b: Appropriate type of mechanism | C3b: The design of the mechanism is appropriate to and can work for the local context (or the national/state context, if it is a national-/state-level mechanism) | The meaning of the question is unclear, and the word ‘design’ is inappropriate | Wording changed to: “The mechanism’s structure and membership is able to influence (e.g. has the power to change or alter) decision-making mechanisms at the level at which it operates (e.g. community, sub-national, state, national)” |
| C3: multi-sectoral, multi-stakeholder | C3d: Structures for interaction between all relevant stakeholders either already existed or were established specifically for this mechanism, e.g. a committee or forum involving all stakeholders that meets regularly | The key point is that the structures should exist – it doesn’t matter whether they existed already or were established by the mechanism. This is not made clear by the wording used. | Wording changed to: ‘d. Structures for interaction between all relevant stakeholders exist, e.g. a committee or forum involving all stakeholders that meets regularly (whether or not they were developed specifically for this mechanism) |
| D2: High-quality monitoring data | D2c: Monitoring data have been independently assessed and found to be accurate | The mechanism itself could be responsible for ensuring accuracy – an independent assessment is not necessary | Wording changed to: ‘Monitoring data have been rigorously assessed and found to be accurate’ |
|  | D2d: Monitoring data are collected in ‘real time’ so they can be reviewed while still recent and relevant | There will nearly always be a time lag – collecting data in ‘real time’ may not be feasible or necessary | Wording changed to: ‘monitoring data are collected on a regular or ongoing basis so they can be reviewed while still recent and relevant’ |
| D3: Solution-focused review of data | D3h: Learning points and decisions taken as a result of the review were communicated clearly to all relevant stakeholders | It should be clarified that this includes those who are external to the mechanism | Wording changed to: ‘Learning points and decisions taken as a result of the review were communicated clearly to all relevant internal and external stakeholders’ |
|  | D3j: The review process yielded documented action plan(s), fully costed and with clear lines of responsibility | This might be overly bureaucratic, especially the ‘fully costed’ element. It’s a review cycle, not a budgeting cycle. | Wording changed to: ‘The review process yielded documented action plan(s) with clear lines of responsibility’ |
| D4: Remedial action in response to review | D4b: There are consequences for duty-bearers who do not implement the parts of the action plan that are their responsibility (e.g. disciplinary procedures, litigation) | May be misinterpreted as contradictory to the ideal philosophy of learning and improving rather than blaming and shaming, as described by criterion D1d | Guidance notes were amended to make it clear that this criterion relates only to duty-bearers who fail to implement actions allocated to them during the review phase of the mechanism |
| F1: Contribution to systemic change | F1c: There have been improvements in health outcomes in the locations where the mechanism was implemented | Some accountability mechanisms target other types of (perhaps intermediary) outcome, e.g. community empowerment, changes to harmful gender norms | Wording changed to: ‘There have been improvements in health or social outcomes in the locations where the mechanism was implemented’, and social outcomes defined in the guidance notes. Participants are asked to provide examples. |
